# N-terminal phosphatase of soluble epoxide hydrolase forms anandamide from anandamide phosphate

**DOI:** 10.1016/j.jbc.2026.113615

**Published:** 2026-09-30

**Authors:** Eriko Suzuki, Hiroki Shidara, Reiji Nagashima, Junki Abe, Ayano Shida, Hitomi Kuwahara, Yuka Uchida, Keiji Hasumi

**Affiliations:** Department of Applied Biological Science, Tokyo University of Agriculture and Technology, Fuchu, Tokyo, Japan

**Keywords:** soluble epoxide hydrolase, phosphatase, vascular endothelial cell, inflammation, anandamide, sphingosine-1-phosphatee, lysophosphatidic acid

## Abstract

Soluble epoxide hydrolase exhibits two distinct activities: C-terminal epoxide hydrolase (C-EH) and N-terminal phosphatase (N-phos). Epoxy fatty acids are the primary substrates of C-EH and exhibit anti-inflammatory properties. N-phos hydrolyses lipid phosphate monoesters, such as isoprenoid pyrophosphates, lysophosphatidic acid (LPA), and sphingosine-1-phosphate (S1P). We found that inhibiting both sEH activities, rather than just C-EH, suppressed vascular endothelial cell (VEC) activation. A negatively charged polar lipid (NCPL) fraction from mouse liver extracts also suppressed VEC activation. This effect was canceled by sEH treatment. The inhibition of isoprenoid and nitric oxide synthesis and the treatment with epoxyeicosatrienoic acid, LPAs, and typical S1P did not inhibit VEC activation, suggesting that a metabolite other than these is responsible for suppressing VEC activation. To identify the sEH substrate responsible for this activity, we performed a non-targeted LC-MS/MS analysis using NCPL. As a result, phospho-*N*-acylethanolamine emerged as a candidate. Enzyme kinetic analysis confirmed that species in this class, including arachidonoyl ethanolamide (anandamide) phosphate, were favorable N-phos substrates. The exogenous addition of anandamide phosphate inhibited VEC activation-associated expression of cell adhesion molecules at concentrations of 3 μM or more.

Mammalian soluble epoxide hydrolase (sEH) has two distinct activities: the C-terminal epoxide hydrolase (C-EH) and the N-terminal phosphatase (N-phos) (1, 2, 3). C-EH activity has been intensively investigated, as its substrates include anti-inflammatory epoxy fatty acids such as epoxyeicosatrienoic acid (EET), which plays a role in various physiological processes (4, 5). N-phos catalyzes the hydrolysis of lipid phosphates such as farnesyl pyrophosphate, lysophosphatidic acids (LPA), and sphingosine-1-phosphates (S1P) (6, 7, 8, 9). Recently, the function of N-phos has been investigated in more detail using inhibitors and genetically edited animals. Inhibition of N-phos modulates vascular endothelial cell (VEC) nitric oxide signaling and vascular endothelial growth factor expression (10, 11, 12), and regulates isoprenoid synthesis and cholesterol transport (13, 14, 15). Animals selectively lacking N-phos exhibit altered energy/fat metabolism and cognitive function (16, 17). However, the molecule that can mediate these actions has not yet been identified.

During the course of our investigation of the inhibitors of N-phos (18, 19), we have found that N-phos inhibition might affect macrophage function through a change in intracellular calcium ion homeostasis (20) and hepatic fibrosis in mice under diabetic conditions (21). In this report, we show that N-phos inhibition suppresses VEC activation *via* a factor independent of LPA, S1P, and the isoprenoid and nitric oxide pathways. A non-targeted LC-MS/MS analysis revealed phospho-*N*-acylethanolamine (p-NAE) as a candidate N-phos substrate. Phospho-arachidonoyl ethanolamide (p-AEA), a precursor for the endocannabinoid arachidonoyl ethanolamide (AEA; anandamide) biosynthesis, was found to be a favorable N-phos substrate and to suppress VEC activation.

## Results

### Inhibition of sEH suppresses VEC activation

When human microvascular endothelial cells (HMVECs) were treated with SMTP-7, an N-phos/C-EH dual inhibitor (22, 23) with significant bioactivities (23, 24, 25), lipopolysaccharide (LPS)-induced VEC activation, as represented by the binding with the monocytoid U937 cells, was significantly reduced compared to nontreated control cells (Fig. 1, *A* and *B*). This effect was seen even when HMVECs were pretreated with SMTP-7 and the agent was washed away before the U937 cell binding assay (Fig. 1*A*, marked with ‘W’). Similarly, other sEH inhibitors relatively selective to N-phos (ebselen (26) and (2*R*)-3-[4-[(2,6-dichlorophenyl)methoxy]phenyl]-2-[(2-methylpropan-2-yl)oxycarbonylamino]propanoic acid; Boc-Tyr(Cl_2_-Bzl) (18)) but not that specific for C-EH (12-[[(tricyclo[3.3.1.13,7]dec-1-ylamino)carbonyl]amino]-dodecanoic acid; AUDA (27)) suppressed VEC activation (Fig. 1, *B* and *C*). The N-phos inhibition upregulates nitric oxide formation (12) and isoprenoid biosynthesis (13). However, the inhibition of nitric oxide synthesis by *N*^G^-nitro-L-arginine methyl ester (L-NAME) or 1400W affected neither the LPS-induction of HMVEC activation nor the effects of SMTP-7 to suppress HMVEC activation (Fig. 1*D*). Similarly, the inhibition of isoprenoid synthesis by statin (pravastatin) (28) did not affect HMVEC activation in the presence or absence of SMTP-7 (Fig. 1*E*).

**Figure 1:**
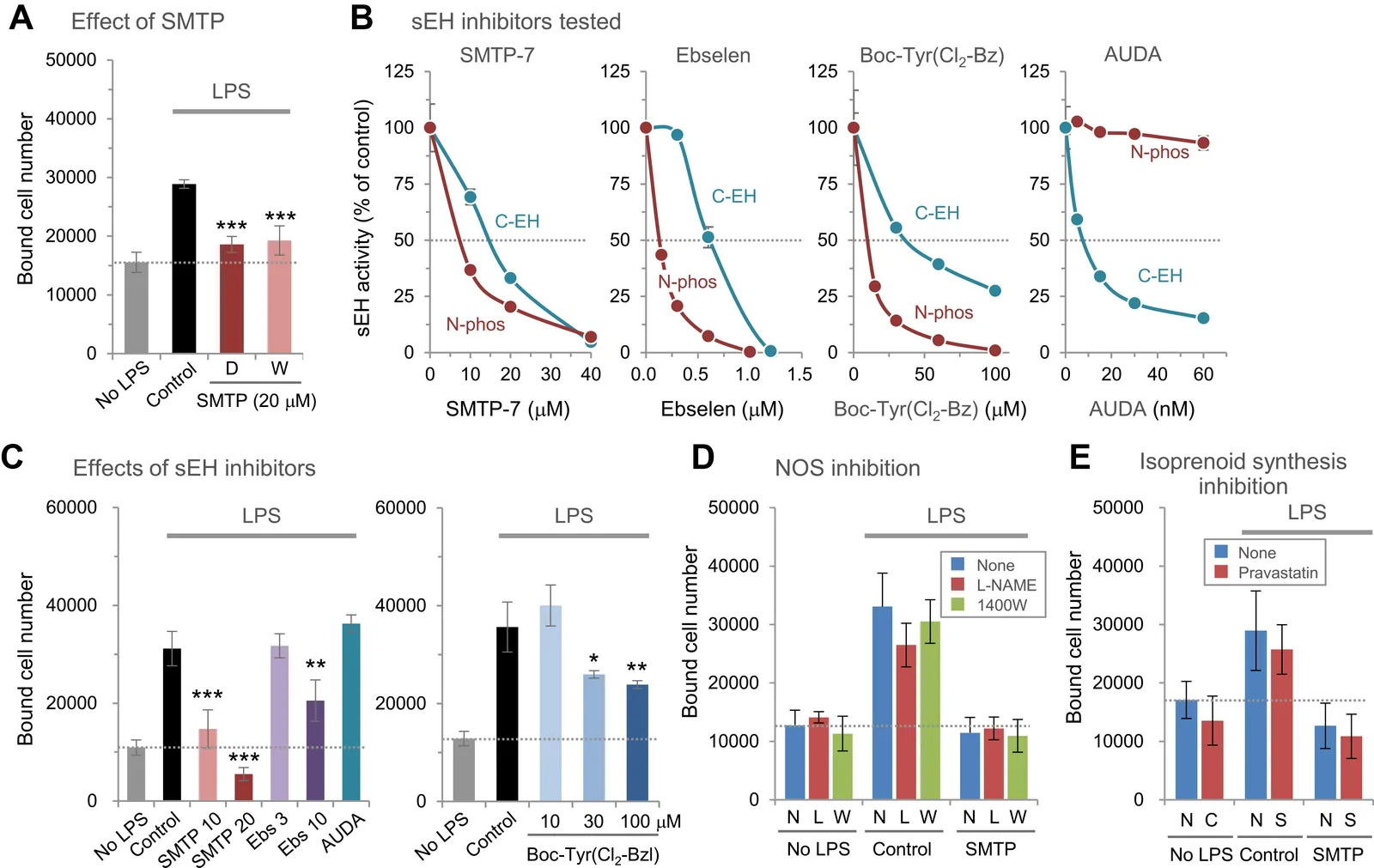
**The sEH inhibition but not the inhibition of the downstream nitric oxide and isoprenoid synthesis suppress VEC activation.** (*A*) Effects of SMTP-7, an N-phos/C-EH dual inhibitor. HMVECs treated with LPS and SMTP-7 were assayed directly for U937 cell binding (denoted as *D*) or after washing (denoted as *W*). (*B*) Inhibition of N-phos and C-EH by the inhibitors used. N-phos activity was determined using the synthetic substrate AttoPhos and C-EH activity using (3-phenyl-oxiranyl)-acetic acid cyano-(6-methoxy-naphthalen-2-yl)-methyl ester (PHOME). (*C*) Effects of SMTP-7, ebselen (Ebs), AUDA, and Boc-Tyr(Cl_2_-Bzl). The concentrations shown are given in μM. (*D*) Effects of nitric oxide synthesis inhibition by L-NAME (1 mM) and 1400W (10 μM) in the presence or absence of SMTP-7 (10 μM). N, nontreated; L-LNAME; W, 1400W. (*E*) Effects of isoprenoid synthesis inhibition by pravastatin (1 μM) in the presence or absence of SMTP-7 (10 μM). N, nontreated; S, pravastatin. Each value represents the mean ± SD from triplicate determinations. ∗*p* < 0.05; ∗∗*p* < 0.01; ∗∗∗*p* < 0.001 by Dunnett test compared with the LPS-treated control.

### N-phos substrate may mediate the suppression of cell adhesion molecule expression in VECs

The inhibition of HMVEC activation by SMTP-7 was associated with the reduction in the expression of intercellular adhesion molecule-1 (ICAM-1), vascular cell adhesion molecule-1 (VCAM-1), and E-selectin (Fig. 2*A*). The effects of other sEH inhibitors were similarly observed with regard to cell adhesion molecule expression (Fig. 2*B*). Consistent with the lack of effect of the C-EH-specific inhibitor AUDA (Fig. 2*B*), the C-EH substrate 14,15-epoxyeicosatrienoic acid (EET) failed to suppress E-selectin expression in HMVECs (Fig. 2*C*). A fraction of negatively charged polar lipids (NCPL) obtained from mouse liver extracts was found to suppress cell adhesion molecule expression, and its activity was diminished by sEH treatment (Fig. 2*D*). Taken together, these results imply that an N-phos substrate may mediate the suppression of VEC activation (Fig. 2*E*). The fact that the VEC activation is not affected by isoprenoid synthesis inhibition and the treatments with LPA and S1P (Fig. S1) suggests that an unknown N-phos substrate, other than isoprenoid pyrophosphates, LPA, and S1P, can mediate the suppression of VEC activation.

**Figure 2:**
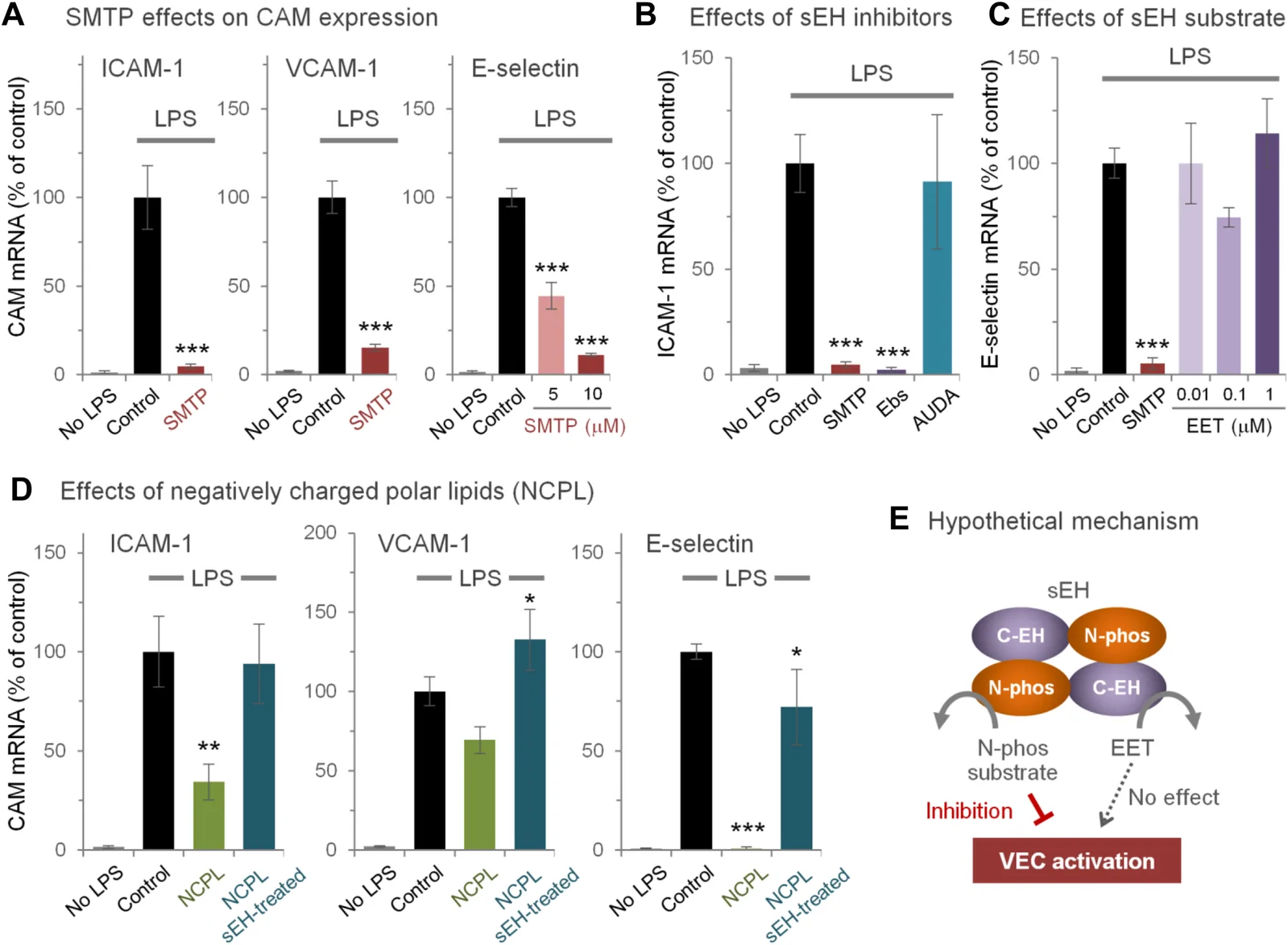
**N-phos substrate may mediate the suppression of cell adhesion molecule expression in VECs.** (*A*) Suppression of the cell adhesion molecule expression by SMTP-7. Unless otherwise shown in the figure, the concentration of SMTP-7 used was 10 μM. (*B*) Differential effects of the N-phos/C-EH dual inhibitors, SMTP-7 (10 μM) and ebselen (Ebs; 10 μM) and the C-EH-specific inhibitor AUDA (5 μM). (*C*) Lack of the effect of the C-EH substrate EET. (*D*) Suppresses of cell adhesion molecule expression by NCPL (10 μg mL^−1^). Its activity was diminished after sEH treatment. (*E*) Hypothetical mechanism suggesting the involvement of an N-phos substrate in the suppression of VEC activation. Each value represents the mean ± SD from triplicate determinations. ∗*p* < 0.05; ∗∗*p* < 0.01; ∗∗∗*p* < 0.001 by Dunnett test compared with the LPS-treated control.

### Exploration of N-phos substrates using a differential labeling method: A targeted LC-MS/MS analysis as a validation

To identify new substrates for N-phos, we conducted a comprehensive LC-MS/MS analysis using NCPL, which might contain a substrate that inhibits VEC activation. The strategy included a differential labeling of the potential N-phos products. sEH-treated and untreated NCPL were labeled with normoisotopic dansyl chloride (DNS-Cl) and deuterated DNS-Cl (DNS(*d*_6_)-Cl), respectively. We then performed an LC-MS/MS analysis targeting several LPA species and S1P to validate this method. In this analysis, potential sEH products can be detected as a pair of ion peaks that differ by six mass units with different intensities (non-substrates should yield a pair of ion peaks with similar intensities) (Fig. 3*A*). As a result, this system identified several species of monoacylglycerol and sphingosine(d18:1) as potential N-phos products (Fig. 3*B* and Table S1). A data-independent, nontargeted analysis using a Thermo Fisher LTQ-Orbitrap spectrometer further confirmed the validity of this method even under nontargeted conditions. Moreover, this analysis revealed atypical sphingosines, sphingosine(d16:1) and sphingosine(d20:1), as N-phos products, the levels of which in mammalian tissues were regarded as less than 1/10 of that of sphingosine(d18:1) (Fig. S2).

**Figure 3:**
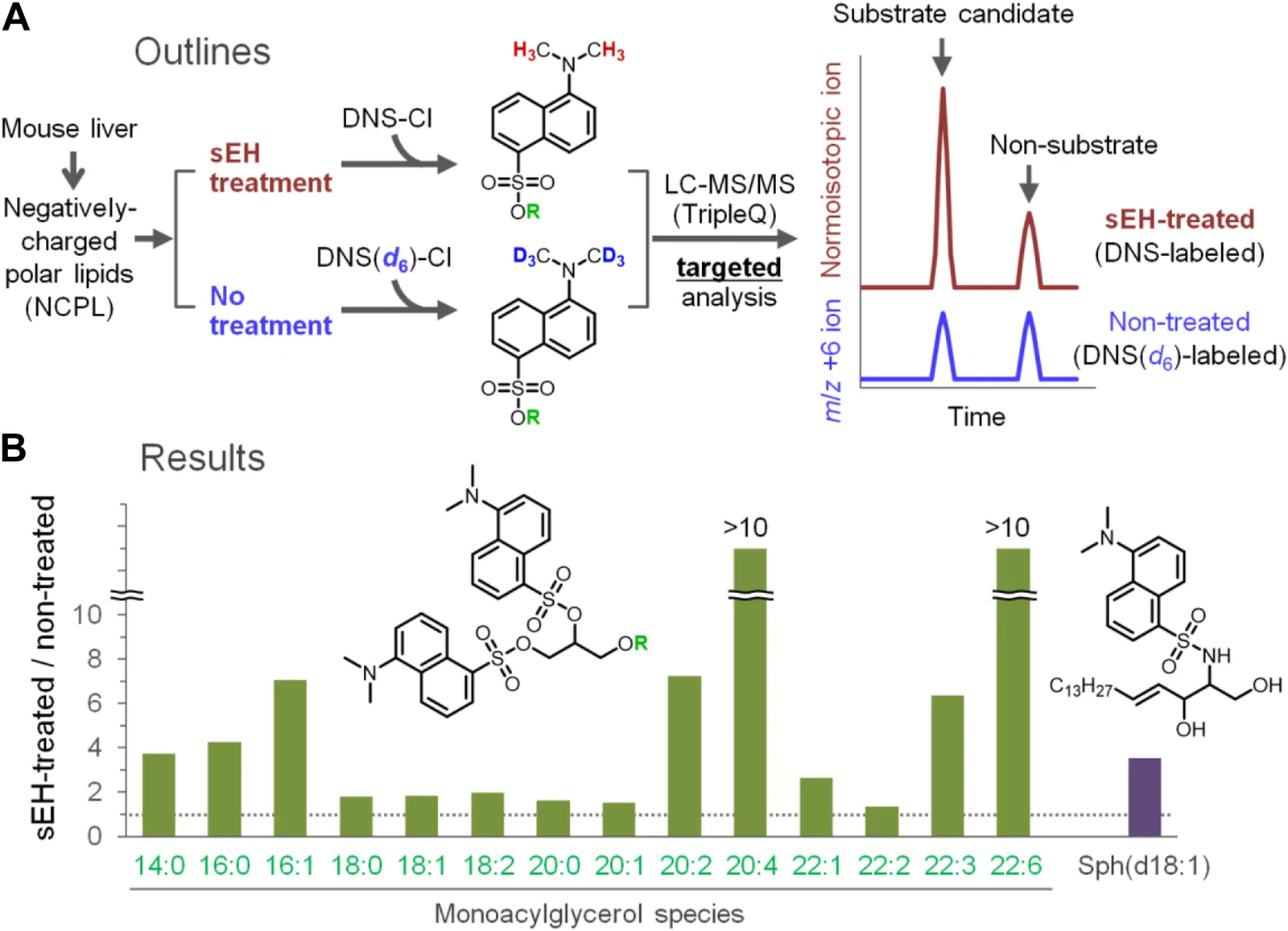
**LC-MS/MS exploration of N-phos substrates using a differential labeling method.** (*A*) Outlines of the experimental strategy in which targeted analysis using a Waters Xevo TQD spectrometer to validate the differential labeling method. The NCPL fraction from mouse liver extracts was differentially labeled with DNS-Cl (normoisotopic) or DNS(*d*_6_)-Cl after sEH treatment or nontreatment, respectively. (*B*) Results of the LC-MS/MS analysis targeted to the products of the representative N-phos substrates LPA and S1P, monoacylglycerol and sphingosine (Sph(d18:1), respectively). The structure of the potential dansyl derivatives is shown. Bar graph shows the relative enrichment after sEH treatment.

### Identification of linoleoyl ethanolamide (LEA) through a nontargeted LC-MS/MS exploration

Further attempts were made to identify the N-phos product using data-independent, nontargeted analysis on a Sciex TripleTOF 6600^+^ spectrometer (Fig. 4*A*). The results suggest that LEA could be a potential candidate for the N-phos product. In the MS/MS chromatogram, the intensity of the normoisotopic product ion at *m*/*z* 170.0964 (for [C_12_H_12_N]^+^) was much higher than that of the deuterated product ion at *m*/*z* 176.1341 (for [C_12_H_6_D_6_N]^+^) (Fig. 4*B*). The mass fragmentation pattern was consistent with the estimated structure, where prominent fragment ions, corresponding to the homolytic fission products from both the dansyl and the LEA moieties, were found (Fig. 4*C*).

**Figure 4:**
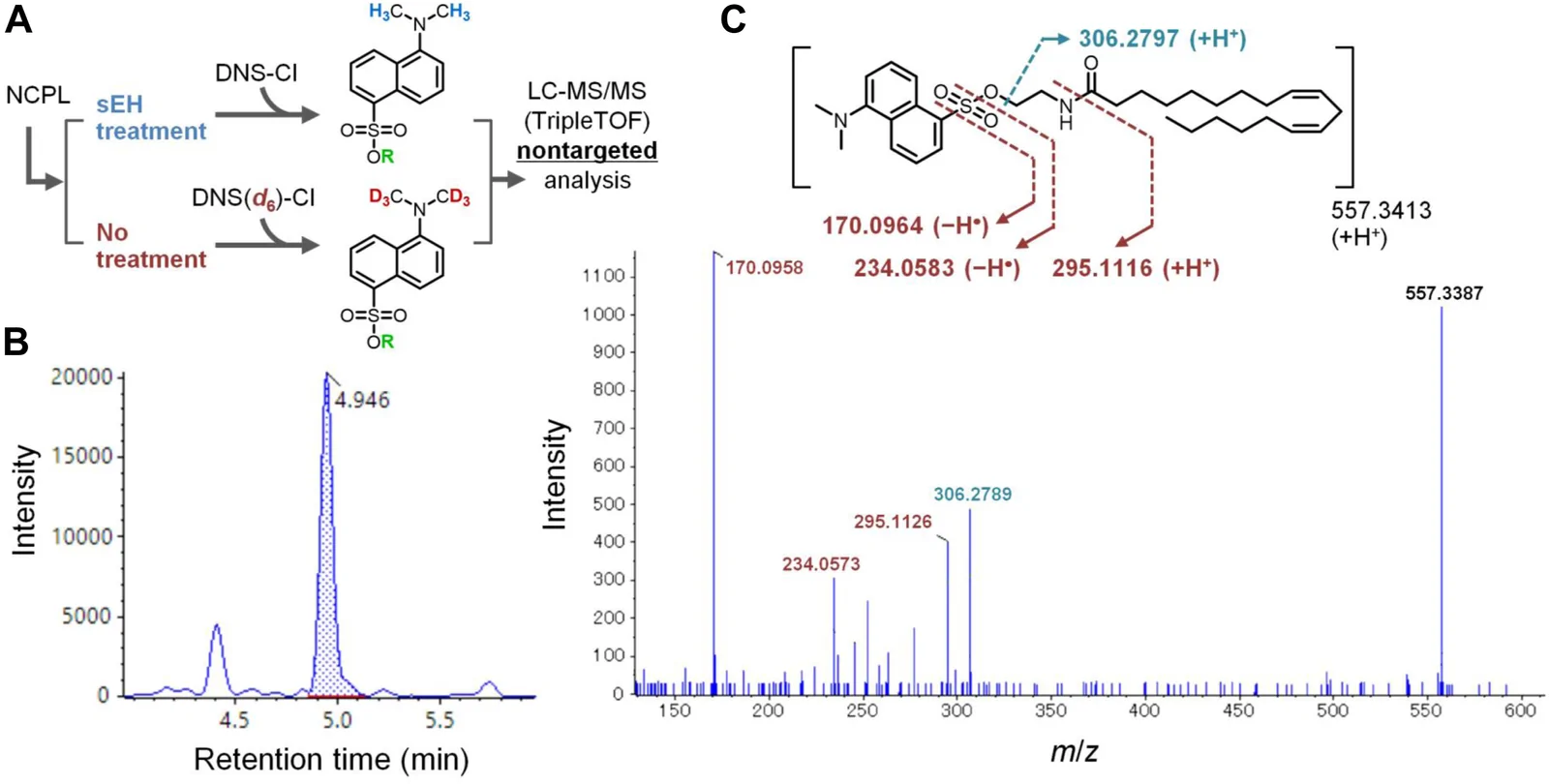
**A nontargeted exploration of N-phos substrates identifies LEA.** (*A*) Outlines of the experimental strategy in which a TripleTOF spectrometer was used to identify trace amounts of unknown N-phos products. (*B*) MS/MS chromatogram. (*C*) Mass spectrum of the potential products. Possible assignments for the daughter ion peaks are shown. These results suggest that the product is LEA.

### p-NAEs are allosteric substrates for N-phos

LEA belongs to a family of bioactive NAEs, which includes AEA (anandamide) (29). One of the major pathways for NAE biosynthesis involves their phosphate ester precursors, p-NAEs (30). To investigate the preference of N-phos for p-NAE substrates, we conducted an enzyme kinetic analysis evaluating representative p-NAE with an oleoyl, a linoleoyl, or an arachidonoyl acyl chain (p-OEA, p-LEA, and p-AEA, respectively). The results indicated that these metabolites were active allosteric substrates for N-phos (Fig. 5). Of the three substrates tested, p-AEA was the most favorable substrate, with a *k*_cat_/*K*_m_ value of 0.42 min^−1^ μM^−1^, which was comparable to the *k*_cat_/*K*_m_ value of 0.53 min^−1^ μM^−1^ for arachidonoyl-LPA (*K*_m_ 53.3 ± 3.22 M^−1^; *k*_cat_ 28.5 ± 1.23; Hill coefficient *n* = 1.5 ± 0.2) obtained in this study. The catalytic efficiencies (*k*_cat_/*K*_m_) of p-LEA and p-OEA were 0.25 and 0.077 min^−1^ μM^−1^, respectively. These results suggest that sEH plays a role in the biosynthesis of NAE.

**Figure 5:**
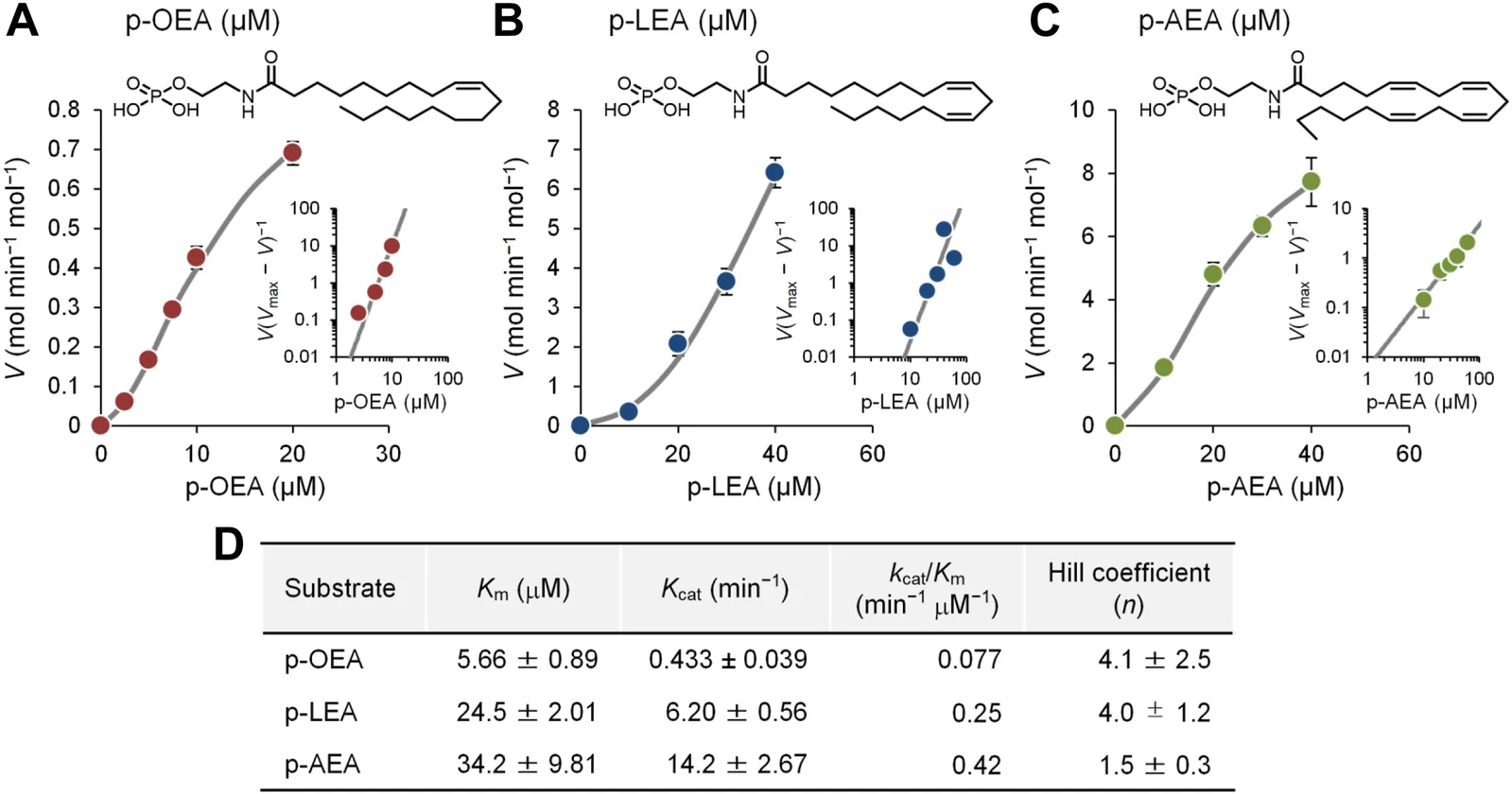
**p-NAEs are allosteric substrates for N-phos.** (*A − C*) Michaelis−Menten and Hill presentations of the enzyme kinetic data for p-OEA, p-LEA, and p-AEA. (*D*) Summary of the kinetic parameters. Each value represents the mean ± SD or SEM (for the kinetic parameters) from triplicate determinations.

### Effects of exogenously added p-NAE on VEC activation

p-OEA and p-LEA significantly inhibited LPS-induced E-selectin expression in HMVECs at 30 μM. Specifically, p-AEA showed significant inhibition at concentrations as low as 10 μM, with 33% and 46% inhibition at 10 and 30 μM, respectively (*p* < 0.001). In addition, p-LEA's effect on ICAM-1 expression was more pronounced, with a significant inhibition at 3 μM and a 68% inhibition at 30 μM (*p* < 0.001) (Fig. 6*B*).

**Figure 6:**
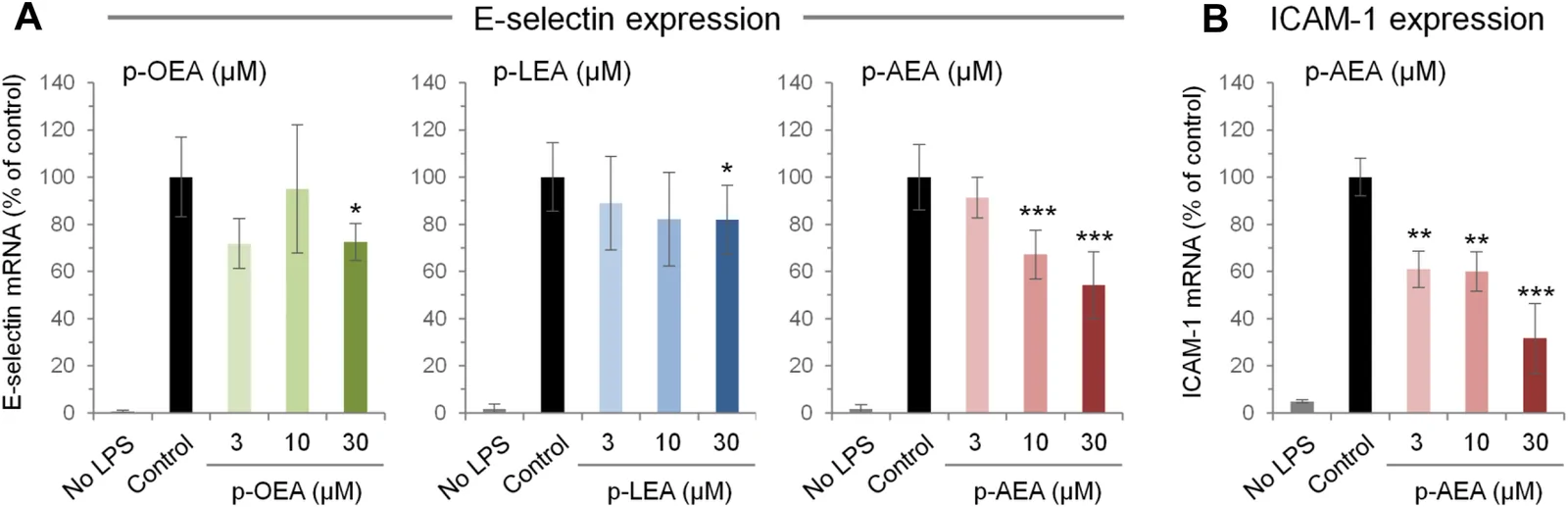
**Effects of exogenously added p-NAE on the VEC activation.** (*A*) Effects of p-OEA, p-LEA, and p-AEA on the LPS-induced E-selectin expression in HMVECs. (*B*) Effects of p-AEA on the LPS-induced ICAM-1 expression in HMVECs. For the E-selectin experiments, each value represents the mean ± SD from two to four experiments, in which each experiment was conducted in triplicate. For the ICAM-1 experiment, each value represents the mean ± SD from triplicate determinations. ∗*p* < 0.05; ∗∗*p* < 0.01; ∗∗∗*p* < 0.001 by Dunnett test compared with the LPS-treated control.

### Levels of p-NAE and NAE in the mouse brain and RAW264.7 macrophage-like cells

Using a newly developed LC-MS/MS method, we determined the endogenous levels of three pairs of p-NAE and NAE (p-OEA/OEA, p-LEA/LEA, and p-AEA/AEA) in the brain of normal wild-type mice and sEH-deficient mice treated with SMTP-7 or vehicle. The brain was selected due to the presence of the p-NAE-to-NAE route (see Discussion for details). Of the three sets of p-NAE/NAE, p-OEA/OEA was present in sufficient amounts (11.3 ± 0.5 and 59.0 ± 6.4 pmol per gram of brain tissue) in wild-type mice (Fig. 7*A*). These values correspond to 14.1 ± 0.6 and 73.8 ± 8.0 nM, respectively, when taking a cellular volume of 0.8 ml per gram of brain tissue. Treatment with SMTP-7 mildly increased the ratio of p-OEA to OEA in wild-type mice (Fig. 7*B*), whereas the difference in the ratio of p-OEA to OEA was not statistically significant between the vehicle control and the SMTP-7-treated group (Fig. 7*C*). sEH-deficient mice exhibited p-OEA and OEA levels like those in wild type mice (Fig. 7*C*). SMTP-7 treatment did not affect the ratio of p-OEA to OEA in sEH-deficient mice (Fig. 7*C*).

**Figure 7:**
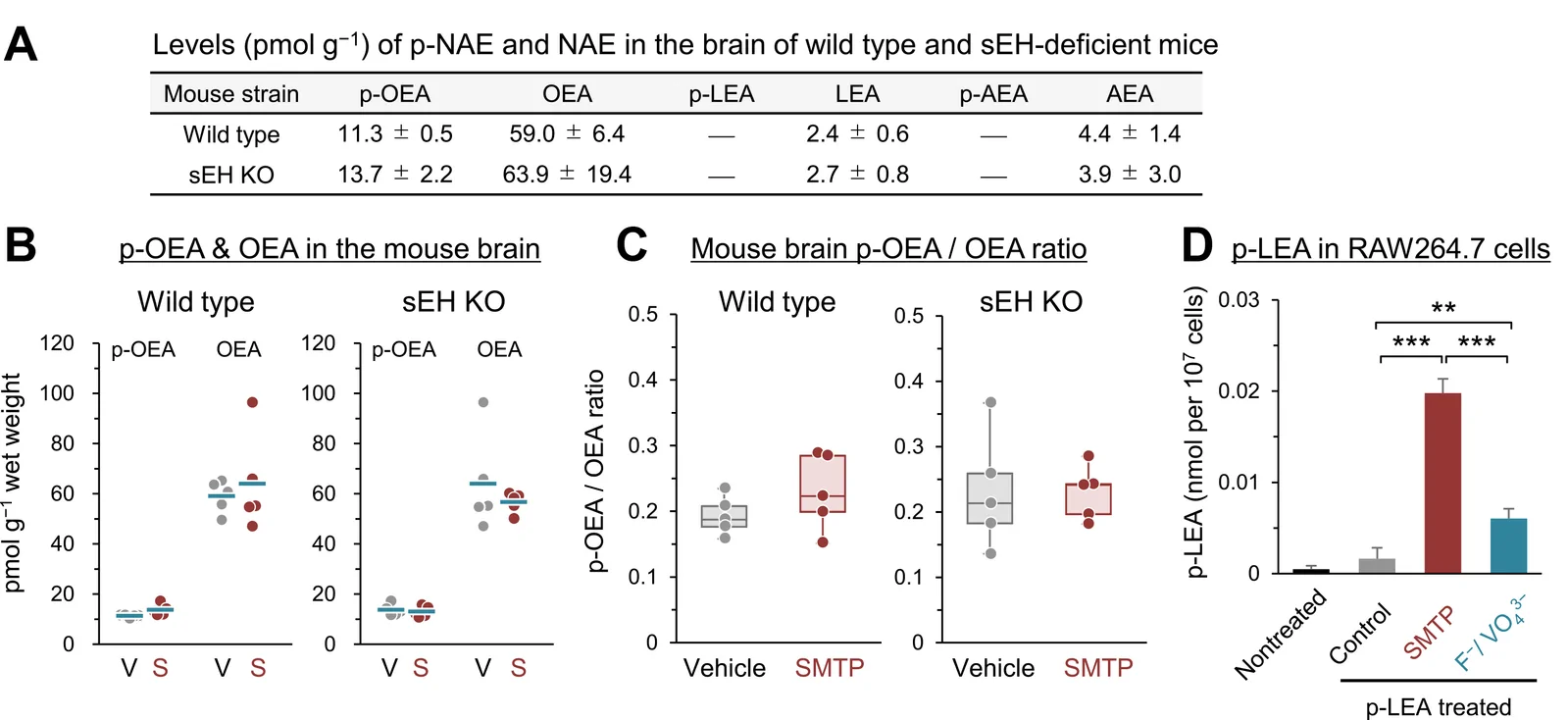
**Levels of p-NAE and NAE in the mouse brain and RAW264.7 cells.** (*A*) Endogenous levels of p-NAE and NAE in the brain of wild-type and sEH-deficient (sEH KO) mice. Each value represents the mean ± SD obtained from the analysis using brain extracts of five animals in each group. —, not determined due to the level being mostly below the limit of detection. (*B*) Levels of p-OEA and OEA in the brain from wild type and sEH-deficient mice treated with vehicle (V) or SMTP-7 (S) at a dose of 20 mg kg^−1^ (*n* = 5 in each group). Each value and the average are shown as a circle and a horizontal bar, respectively. (*C*) The ratio of p-OEA to OEA derived from the data presented in *panel B*. Box plots with the median and the interquartile range are shown. Whisker represents the maximum and minimum within 1.5 times of the interquartile range. Individual values are overlayed on box plots. Data were derived from five animals in each group. Regarding p-OEA/OEA ratio, statistical analysis using Kruskal-Wallis test suggested that the difference between the vehicle and SMTP groups was not significant (*p* = 0.35 for wild type mice and *p* = 0.92 for eEH deficient mice). (*D*) Levels of p-LEA in RAW264.7 cells after treatment without (endogenous) or with exogenous p-LEA (30 μM) for 15 min. In the p-LEA treatment experiments, cells were simultaneously treated with vehicle (control), 20 μM SMTP-7, or a mixture of 5 mM NaF and 400 μM Na_3_VO_4_ (F^−^/VO_4_^3−^). Each value represents the mean ± SD from five (endogenous level) or three replicates (levels following exogenous p-LEA addition). ∗∗*p* < 0.01; ∗∗∗*p* < 0.001 by Tukey-Kramer test compared with each of the groups. Key differences are shown.

We also assessed the levels of p-NAE and NAE in RAW264.7 cells (Fig. 7*D*). The endogenous levels of p-OEA, OEA, LEA, p-AEA, and AEA were below the limit of detection, whereas the p-LEA level was detectable. The value was 0.48 ± 0.38 fmol per 10^7^ cells. This value corresponds to 9.7 ± 7.5 nM when taking the cellular volume as 5 pL per cell. Treatment of RAW264.7 cells with 30 μM of exogenous p-LEA for 15 min elevated the cellular p-LEA level to 1.6 ± 1.2 fmol per 10^7^ cells (33 ± 24 nM). When SMTP-7 and a mixture of fluoride and vanadate were added simultaneously, the cellular p-LEA level increased by ∼12 and 3.7 times, respectively. These results suggest that sEH regulates p-LEA level in RAW264.7 cells.

## Discussion

This study reveals the following five points: (i) sEH inhibition results in the suppression of VEC activation; (ii) N-phos substrate(s) may play a role in this effect; (iii) a nontargeted exploration identifies a species of NAE as a candidate N-phos substrate; (iv) N-phos dephosphorylates p-NAEs to form NAEs such as anandamide; and (v) treatment of VECs with p-NAEs, particularly p-AEA, results in the suppression of VEC activation.

The treatment with N-phos/C-EH dual inhibitors suppressed VEC activation, whereas the C-EH inhibitor had no effect. These results raise the possibility that N-phos inhibition plays a role in this suppression. N-phos inhibition is implicated in the control of nitric oxide formation (12) and isoprenoid synthesis (13), suggesting an involvement of nitric oxide or isoprene pyrophosphates in the suppression of VEC activation. However, the inhibition of the synthesis of nitric oxide or isoprene pyrophosphates does not affect VEC activation. Therefore, the regulation of VEC activation by sEH may not require the modulation of nitric oxide and isoprenoid biosynthetic pathways. The representative C-EH substrate, EET, has no effect on VEC activation, whereas the fraction of acidic phospholipid NCPL suppressed VEC activation. The activity of NCPL is diminished by sEH treatment. These results suggest that an N-phos substrate may mediate the suppression of VEC activation. Given that isoprenoid biosynthesis does not affect VEC activation, an N-phos substrate other than isoprenoid pyrophosphates may participate in the suppression of VEC activation. Moreover, the failure of LPA species and typical S1P to suppress VEC activation supports this idea.

Using a differential labeling method for the detection of potential N-phos substrates, a nontargeted LC-MS/MS analysis identifies a species of NAE as a candidate. The validity of this method is confirmed by the fact that this method identifies several LPA species and typical S1P. NAEs are a family of bioactive fatty acid ethanolamides with differing fatty acyl moieties, such as oleoyl, linoleoyl, and arachidonoyl (29). The reason why we fail to detect NAE species other than LEA may be related to their abundance. Therefore, increasing sensitivity can enable the detection of minor NAE species as well as other types of N-phos substrate.

We demonstrate that the p-NAE species p-OEA, p-LEA, and p-AEA act as N-phos substrates that form corresponding NAEs. Like other substrates (6, 9, 31), these are allosteric substrates with Hill coefficients ranging from 1.5 to 4.1. From the viewpoint of catalytic efficiency, p-AEA is a favorable N-phos substrate comparable to arachidonoyl LPA. The product NAEs are signaling lipids exhibiting various physiological activities (29, 30). For example, palmitoyl ethanolamide is anti-inflammatory and analgesic; oleoyl ethanolamine is anorectic and anti-inflammatory; LEA is anti-inflammatory and regulatory to metabolism; and AEA acts as an endogenous cannabinoid neurotransmitter and modulates pain and inflammation (29). NAE biosynthesis involves four routes stemming from the intermediate *N*-acylphosphatidylethanolamine, which is formed from phosphatidylethanolamine *via* acyl transfer from the *sn*-1 position of another phospholipid such as phosphatidylcholine (Fig. 8) (30). p-NAE is an intermediate positioned in one of the four routes. This route is considered a noncanonical pathway that operates in the brain and activated macrophages (32, 33) and may be involved in osteoarthritis development (34). The phosphatases protein tyrosine phosphatase non-receptor type 22 (PTPN22) and SH2 domain-containing inositol polyphosphate 5-phosphatase 1 (SHIP1) are known to convert p-NAE to NAE (32, 33). This study suggests that sEH is an additional contributor to this route.

**Figure 8:**
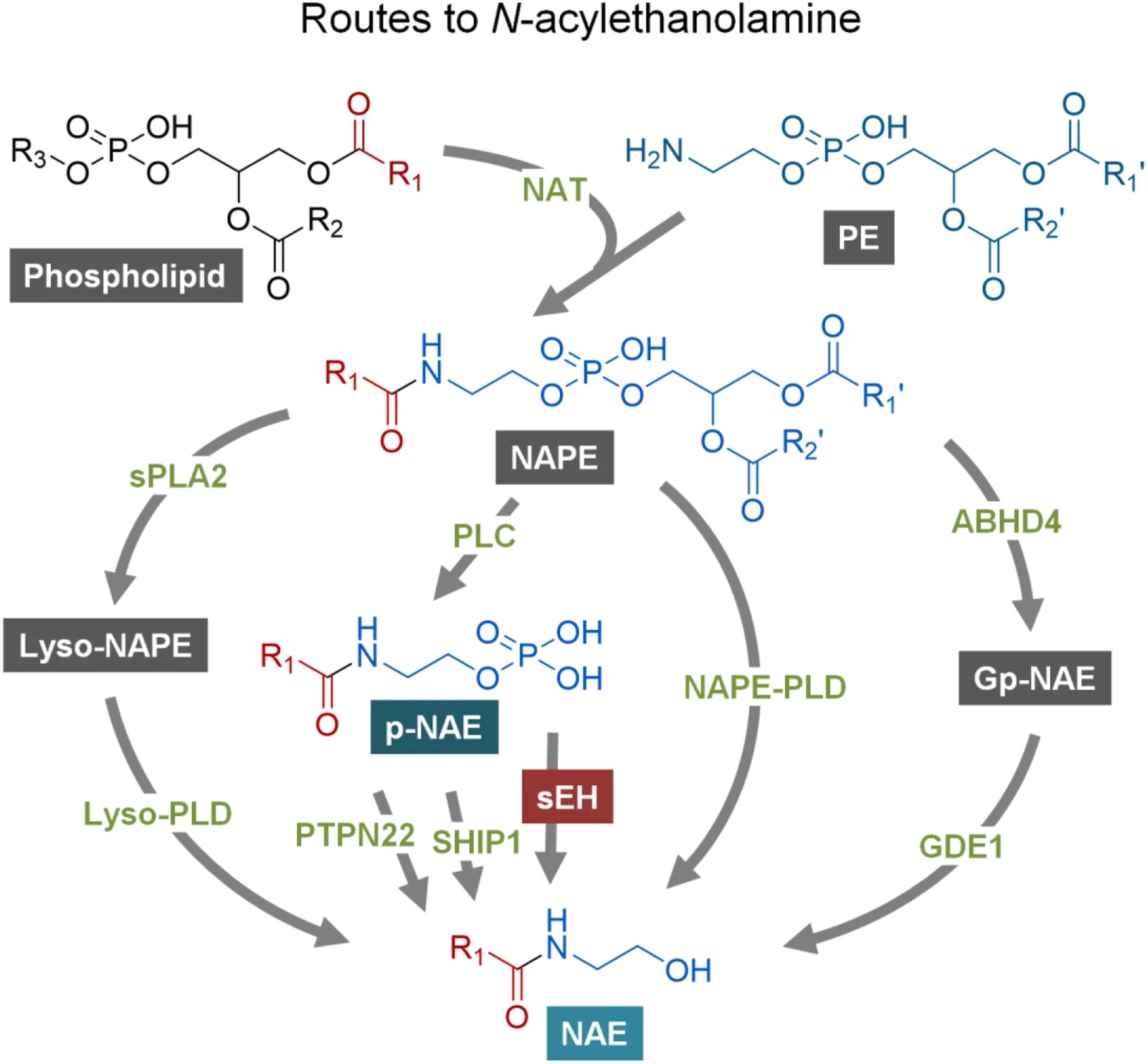
**Possible role of sEH in NAE biosynthesis.** NAE is formed from the precursor *N*-acylphosphatidylethanolamine (NAPE), which is synthesized from phosphatidylethanolamine (PE) and a phospholipid such as phosphatidylcholine. Four routes to NAE formation from NAPE have been reported. One is catalyzed by NAPE-specific phospholipase D (NAPE-PLD), which directly yields NAE. Other routes involve an intermediate in each: lyso-NAPE, p-NAE, or glycerophospho-NAE (Gp-NAE). In the route involving the p-NAE intermediate, the role of the phosphatases PTPN22 and SHIP1 have been characterized. This study suggests that sEH is an additional contributor to this route. NAT, *N*-acyltransferase; sPLA2, secreted phospholipase A2; PLC, phospholipase C; ABHD4, abhydrolase domain containing 4; lyso-PLD, lysophospholipase D (autotaxin); GDE1, glycerophosphodiester phosphodiesterase 1.

We have determined the endogenous levels of p-NAE and NAE in the mouse brain and obtained OEA levels comparable to the values reported previously (35, 36). Unfortunately, we are unable to compare our p-OEA data with that in the literature, as this information is not available. p-OEA levels are not statistically significantly elevated in the brains of wild-type mice following SMTP-7 treatment. The presence of the abovementioned pathways for the conversion of p-NAE to NAE in the brain may prevent a clear increase in the p-NAE to NAE ratio following sEH inhibition. Similarly, levels of phospholipids including LPA and S1P in the liver of a mouse line lacking functional N-phos activity (D9A point mutation introduced at the catalytic site in the N-phos domain using the CRISPR-Cas9 technology; Hatada I, Horii T, Suzuki E, Hasumi K, unpublished results) are comparable to wild-type animals. Thus, compensatory mechanisms may have obscured the effects of the genetic absence of N-phos activity. This idea is also consistent with the finding that the level of p-OEA in the brains of sEH-deficient mice is not higher than in wild-type mice.

On the other hand, p-LEA-treated RAW264.7 cells, which possess the p-NAE-to-NAE route like the brain, exhibit a significantly elevated level of cellular p-LEA after SMTP-7 treatment. The difference may arise from variations in the activity of PTPN22 and SHIP1 in the p-NAE-to-NAE pathway compared to sEH activity. It should be noted that sEH is not sensitive to fluoride and vanadate (2), whereas PTPN22 is highly sensitive to vanadate (37), which is less effective in increasing p-LEA in p-LEA-treated RAW264.7 cells compared to SMTP-7.

Treatment of HMVECs with p-NAEs, particularly pAEA, results in a decrease in VEC activation. However, the concentrations required for this effect are relatively high (in a μM range). Although p-NAE levels are not available in the literature, the physiological NAE levels are reported to be in the range of nM or nmol kg^−1^ of tissue (38, 39). In our experiment, p-NAEs are added exogenously as a solution in DMSO, and we cannot tell what proportion of the p-NAE is incorporated into the cell. Therefore, we tried to determine endogenous p-NAE using the same method used for the p-NAE determination in RAW264.7 cells. Endogenous p-NAE levels in HMVECs were all below the detection limit. Cellular p-NAE increased following incubation with 30 μM of p-LEA, but the values varied considerably between incubations. Therefore, it was not possible to evaluate the effects of the sEH inhibitor on the cellular p-NAE level in HMVECs. This prevented us from unambiguously delineating the role of p-NAEs in regulating VEC activation. We therefore note this as a significant limitation of the study that should be addressed in future research. In addition, we do not address the mechanism of the p-NAE suppression of VEC activation in this study, and further investigations are warranted to understand details and significance of the p-NAE action.

## Experimental procedures

### Materials

Recombinant human sEH (N-terminal His-tagged protein; item No. 10011669) was purchased from Cayman Chemical. The synthetic N-phos substrate AttoPhos was obtained from Promega, and the C-EH substrate PHOME from Cayman Chemical. SMTP-7 was isolated as described previously (40). Boc-Tyr(Cl_2_-Bz) was a product of TCI, and L-NAME, 1400W, and pravastatin of Sigma-Aldrich. DNS-Cl and DNS-Cl-*d*_6_ were obtained from TCI and Toronto Research Chemicals, respectively. The following chemicals were purchased from Cayman Chemicals: AUDA, ebselen, 14,15-epoxyeicosatrienoic acid (14,15-EET), LPA(16:0), LPA(18:1), LPA(20:4), stearoyl ethanolamide, S1P, heptadecanoyl ethanolamide (HEA), oleoyl ethanolamide (OEA), p-OEA, LEA, p-LEA, AEA, and p-AEA. Phospho-heptadecanoyl ethanolamide (p-HEA) was purchased from Avanti Research (Alabaster, AL, USA). Unless otherwise indicated, all the compounds were assayed as DMSO solutions at the final DMSO concentrations of 0.3% (vol vol^−1^) or less. HMVECs were obtained from Cell Applications, and U937 cells and RAW264.7 macrophage-like cell line were provided by the Institute of Development, Aging and Cancer, Tohoku University.

### U937 cell binding to HMVECs

HMVECs seeded at 1.5 × 10^4^ cells in a 24-well plate for 24 in CADMEC medium (Cell Applications) were treated with LPS (1 μg mL^−1^) for 3.5 h. Compounds tested were included during the LPS treatment. Subsequently, HMVECs received a suspension (1.0 × 10^5^) of U937 cells, which were labeled with 0.7 μg mL^−1^ of 5(6)-carboxyfluorescein diacetate *N*-succinimidyl ester (DOJINDO, Kumamoto, Japan) for 15 min and washed twice with phosphate-buffered saline (PBS). Unbound U937 cells were removed by washing twice with PBS. HMVEC-bound U937 cells were lysed in 0.2% (wt vol^−1^) SDS, and fluorescence (516 nm with an excitation at 495 nm) of the lysate was determined after centrifugation to remove insoluble materials.

### Cell adhesion molecule expression in HMVECs

HMVECs seeded at 1.5 × 10^4^ cells in a 24-well plate were pretreated for 30 min with an agent to be tested (dissolved in DMSO; the final DMSO concentrations was kept at 0.3% (vol vol^−1^) or less). Subsequently, cells were treated with LPS (1 μg mL^−1^) for 3 h. After incubation, total RNA was extracted using RNAiso Plus (Takara Bio, Kusatsu, Japan) and assayed for real-time quantitative reverse-transcription PCR using a PrimeScript One Step RT-PCR Kit 2 (Takara Bio) or equivalent. The primer sets used were shown in Table S2.

### Assay for N-phos using AttoPhos as a substrate

To a 96-well black plate, 60 μl of buffer A (25 mM Bis-Tris-HCl, pH 7.0, 1 mM MgCl_2_, and 0.1 mg mL^−1^ bovine serum albumin) containing 60 ng of sEH and 20 μl of test sample solution of any concentration diluted with buffer A were added for 5 min. Subsequently, 20 μl of 25 μM AttoPhos in buffer A was added. The reaction was stopped by adding 50 μl of 10 mM NaOH after incubation at 37 °C for 90 min. Fluorescence intensity at excitation and emission wavelengths of 450 nm and 545 nm, respectively, was measured.

### Assay for N-phos using natural substrates

Buffer A containing sEH (40 nM) and the substrate to be tested were incubated at 37 °C for 30 min. Subsequently, the reaction was stopped by adding 450 μl of acetonitrile. Then, 10 μl of stearoyl ethanolamide (330 nM in methanol) was added as an internal standard. The mixture was centrifuged at 20,000×*g* for 5 min at 25 °C to obtain the supernatant, which was then subjected to LC-MS/MS analysis of the product (see below: LC-MS/MS for the assay for the N-phos enzyme assay to determine the conversion of p-NAE to NAE). Data were normalized for the recovery of the internal standard stearoyl ethanolamide.

### Assay for C-EH

C-EH activity was determined using PHOME as a substrate. To a 96-well black plate, 60 μl of buffer A containing 60 ng of sEH enzymes and 20 μl of effector solution of any concentration diluted with buffer A were added. The reaction was started by adding 20 μl of 25 μM PHOME in buffer A. Fluorescence intensity at 465 nm (with excitation at 330 nm) was measured kinetically at 37 °C for 30 min.

### Lipid extraction and purification

The following animal experiment was conducted after approval by the Animal Experiment Subcommittee, Tokyo University of Agriculture and Technology (approval No. R03-97): Male ICR mice obtained from Sankyo Labo Service (Tokyo, Japan) received an intravenous injection of the N-phos/C-EH dual inhibitor SMTP-7 (20 mg kg^−1^) to accumulate potential substrates for sEH. One hour after the injection, mice were sacrificed to remove the liver, which was then homogenized in methanol to extract polar lipids containing lysophospholipids and phospholipids (41). Briefly, 1 g of the liver was homogenized in 5 ml of water. The homogenates were mixed with 75 ml of methanol for 10 min. The mixture was centrifuged at 1500×*g* for 5 min to obtain supernatant, which was then evaporated to dryness. The residue was homogenized in *n*-hexane at 10 mg mL^−1^ to obtain supernatant by centrifugation at 2000×*g* for 2 min. The resulting pellets were extracted again with the half volume of *n*-hexane. The combined extracts were evaporated to dryness. The residue was dissolved in chloroform at 5 mg mL^−1^, and 30 ml of the extract was applied to a DEAE-cellulose column (acetate form; 750 mg) equilibrated with chloroform. The column was successively developed with 60 ml of the following solvents: (i) chloroform, (ii) chloroform−methanol (1:1), and (iii) chloroform−methanol−28% (wt wt^−1^) ammonia water−methanol (200:100:3:0.9). Step (i) removes neutral lipids; step (ii) removes polar lipids without a negative charge; and step (iii) elutes polar lipids with a negative charge, NCPL.

### sEH treatment of NCPL

NCPL emulsified in a buffer consisting of 25 mM MOPS and 1 mM MgCl_2_, pH 7.0, at 0.5 mg mL^−1^ was incubated at 37 °C for 24 h with mouse sEH (22) at a final concentration of 26 μg mL^−1^. As a nontreated control, NCPL was incubated without the enzyme. The reaction was stopped by adding formic acid (2 μL mL^−1^). The mixture (1 ml) was extracted thrice with 1 ml of toluene after the addition of 200 mg of NaCl. The toluene extracts were dried over anhydrous sodium sulfate and evaporated to dryness. The residue was dissolved either in DMSO for VEC activation assays or in dichloromethane for derivatization. We conducted the lipid extraction/purification and sEH treatment experiments twice and obtained reproducible results, including the mass spectrometry described below.

### Differential labeling of NCPL

sEH-treated or nontreated NCPL prepared as above was dissolved in 0.5 ml of dichloromethane containing 4-dimethylaminopyridine (10 mg mL^−1^). The solution was mixed with 0.25 ml of dichloromethane containing *n*-hexadecanol (2.8 μg mL^−1^) and *N*,*N*-diisopropylethylamine (5%, vol vol^−1^). Finally, the mixture received 0.5 ml of dichloromethane containing either DNS-Cl or DNS(*d*_6_)-Cl (2.8 μg mL^−1^). The mixture was incubated at 65 °C for 1 h and evaporated to dryness. The residue was suspended with brine (1 ml), extracted thrice with toluene (1 ml), dried over anhydrous sodium sulfate, and evaporated to dryness. The residue was dissolved in acetonitrile (0.2 ml) and filtered using a 0.45 μm polytetrafluoroethylene filter for LC-MS/MS analysis. *n*-Hexadecanol was included in this reaction to monitor the efficiency of the derivatization with DNS-Cl.

### LC-MS/MS for N-phos substrate identification

The instruments used and the analytical conditions are summarized in Table S3 (systems 1, 2, and 3). The protocol for the targeted analysis (system 1 in Table S3) was for the monitoring of the precursor ions as well as for exploratory multiple reaction monitoring of daughter ions derived from the dansyl moiety. The protocols for the nontargeted analysis (systems 2 and 3 in Table S3) employed a multiple reaction monitoring mode intended to primarily detect daughter ions from the dansyl moiety, taking that the dansyl adducts yielded characteristic ions such as [C_12_H_12_N]^+^ at *m*/*z* 170.0964 (for the normoisotopid dansyl derivatives) and [C_12_H_6_D_6_N]^+^ at *m*/*z* 176.1341 (for the deuterated dansyl derivatives) (see Figs. 2A and 4). Ion peak intensity was normalized using a dansyl derivative of n-hexanol, which was added to NCPL prior to dansylation.

### LC-MS/MS for the assay for the N-phos enzyme activity to determine the conversion of p-NAE to NAE

The products of the N-phos reaction were quantified under the conditions summarized in Table S3 (system 4), where multiple reaction monitoring was used. Data were normalized using the value of the internal standard stearoyl ethanolamide added after the enzyme reaction. The amounts of the products, OEA, LEA, and AEA, were calibrated using authentic samples.

### Endogenous levels of p-NAE and NAE in the mouse brain

The following animal experiment was conducted after approval by the Animal Experiment Subcommittee, Tokyo University of Agriculture and Technology (approval No. R06-145): Male C57BL/6NCrslc mice (Sankyo Labo Service, Tokyo, Japan), and male sEH-deficient mice (B6.129X-*Ephx2*^*tm1Gonz*^/J, Jackson Laboratory, Bar Harbor, ME, USA) were given intraperitoneal dose of PBS or SMTP-7 (20 mg kg^−1^) at the age of 18 weeks. After 3 h, mice were sacrificed to remove the brain, which was then homogenized in nine volumes of acidic methanol (methanol supplemented with 5 mM ammonium formate and 0.1%, v v^−1^, formic acid) containing 5 μM of each of the p-HEA and HEA internal standards. The homogenates were centrifuged at 2200×*g* for 10 min at 4 °C to obtain the supernatant. The resulting extracts were concentrated to dryness and dissolved in 1/20 volume of acidic methanol. The resulting samples were centrifuged at 20,630×*g* for 10 min at 4 °C, and the supernatants were analyzed by LC-MS/MS as described below.

### Levels of p-NAE and NAE in RAW264.7 cells

For the determination of endogenous levels of p-NAE and NAE, subconfluent RAW264.7 cells grown on 100-mm dishes in RPMI-1640 were washed three times with ice-cold PBS, scraped off the dishes, and centrifuged at 300×*g* for 10 min at 4 °C. The resulting cell pellets were extracted with acidic methanol containing 5 μM of each of the p-HEA and HEA internal standards by sonication for 1 min (2 ml for cells recovered from each of the 100-mm dish). The resulting extracts were concentrated to dryness and dissolved in 1/20 volume of acidic methanol. The resulting samples were centrifuged at 20,630×*g* for 10 min at 4 °C, and the supernatants were analyzed by LC-MS/MS as described below.

For the analysis of the uptake of p-NAE, subconfluent RAW264.7 cells grown on 100-mm dishes in RPMI-1640 were pretreated with PBS, SMTP-7 (20 μM), or a mixture of NaF (5 mM) and Na_3_VO_4_ (400 μM) for 30 min at 37 °C. Subsequently, the medium was replaced with one containing the same additives and p-LEA (30 μM). After incubation at 37 °C for 15 min, cells were processed for LC-MS/MS analysis as described for the determination of the endogenous levels of p-NAE and NAE.

### LC-MS/MS for the determination of p-NAE and NAE in the mouse brain and RAW264.7 cells

p-OEA, OEA, p-LEA, LEA, p-AEA, and AEA were quantified under the conditions summarized in Table S5 (system 5), where multiple reaction monitoring was used. Data were normalized using the value of the internal standards p-HEA and HEA. The amounts of the metabolites were calibrated using authentic samples.

### Data analysis

Enzyme activity is expressed as mol of substrate hydrolyzed per min per mol of enzyme (mol min^−1^ mol^−1^). For the kinetic analysis, the concentration of the substrate was changed as shown in each figure. Data were analyzed using SigmaPlot 14.5 (Inpixon). Since all the data obtained appeared to fit the Hill equation, the kinetic parameters *K*_m_, *V*_max_, *k*_cat_, and Hill coefficient (*n*) were determined by regression analysis based on the Hill equation.

## Data availability

The data presented in this study are available in this article and supplementary material.

## Supporting information

This article contains supporting information.

## Conflicts of interest

The authors declare the following financial interests/personal relationships which may be considered as potential competing interests:

The authors other than K.H. declare no conflict of interest. K.H. is a Director of the Board and shareholder of TMS Co., Ltd, one of the funders, which had no role in the design of the study; in the collection, analysis, or interpretation of data; in the writing of the manuscript, or in the decision to publish the results.
