# Supplementary material for: N-terminal phosphatase of soluble epoxide hydrolase forms anandamide from anandamide phosphate
Source: J Biol Chem. 2026 Sep 30;302(11):113615. doi: 10.1016/j.jbc.2026.113615 (PMC13737751; doi:10.1016/j.jbc.2026.113615)
Supplement: Supplementary file 1 [file mmc1.pdf]

Supporting Information for:

N-terminal phosphatase of soluble epoxide hydrolase forms anandamide from anandamide phosphate

Eriko Suzuki [1]*, Hiroki Shidara [1], Reiji Nagashima [1], Junki Abe [1], Ayano Shida [1], Hitomi Kuwahara [1], Yuka Uchida [1], and Keiji Hasumi [1,]*

[1] Department of Applied Biological Science, Tokyo University of Agriculture and Technology, 3-5-8 Saiwaicho, Fuchu, Tokyo 183-8509, Japan

* Correspondence: ersuzuki@cc.tuat.ac.jp; hasumi@cc.tuat.ac.jp

Table S1. Results of the targeted LC-MS/MS analysis

Table S2. Primers for real time quantitative reverse-transcription PCR

Table S3. LC-MS/MS conditions

Figure S1. LPA and S1P lack the activity to suppress HMVEC activation

Figure S2. A nontargeted exploration of N-phos substrates identifies minor S1P species

Table S1. Results of the targeted LC-MS/MS analysis

Assignment	Non-treated (DNSd_6-labeled)				sEH-treated (DNS-labeled)				Ratio
	t_R (min)	*m/z* calcd	*m/z* found	Area	t_R (min)	*m/z* calcd	*m/z* found	Area	
$[MG\text{-}(DNS)_2]^{2+}$									
14:0	5.79	781.4305	781.43	133	5.83	769.3551	769.36	497	3.7
16:0	6.13	809.4618	809.46	1703	6.15	797.3864	797.39	7267	4.3
16:1	5.83	807.4461	807.44	61	5.84	795.3708	795.37	431	7.1
18:0	6.46	837.4931	837.49	3001	6.50	825.4177	825.42	5447	1.8
18:1	6.12	835.4774	835.48	1291	6.15	823.4021	823.40	2375	1.8
18:2	5.85	833.4618	833.46	1222	5.87	821.3864	821.39	2405	2.0
20:0	8.13	865.5244	865.55	9921	8.17	853.4490	853.45	16327	1.6
20:1	7.58	863.5087	863.52	33643	7.61	851.4334	851.43	50950	1.5
20:2	7.15	861.4931	861.51	152	7.20	849.4177	849.42	1095	7.2
20:4	—	857.4618	—	—	5.16	845.3864	845.39	712	>10
22:1	8.67	891.5400	891.56	1847	8.69	879.4647	879.46	4877	2.6
22:2	7.64	889.5244	889.55	1648	7.66	877.4490	877.45	2211	1.3
22:3	7.39	887.5087	887.52	81	7.40	875.4334	875.43	518	6.4
22:6	—	881.4618	—	—	5.69	869.3864	873.42	1030	>10
$[Sph\text{-}DNS]^{+}$	5.80	539.3785	539.38	6184	5.82	533.3408	533.3400	21730	3.5

t_R, retention time; calcd, calculated; Sph, sphingosine; —, below the lower limit of quantification

Table S2. Primers for real time quantitative reverse-transcription PCR

Target gene	Nucleotide sequence	
E-selectin	Sense	5'- GGTTGAGTGTGATGCTGTGA -3'
	Antisense	5'- GAAGGTGAACTCTCCAGCAG -3'
ICAM-1	Sense	5'- GGCTGGAGCTGTTTGAGAAC -3'
	Antisense	5'- TCACACTGACTGAGGCCTTG -3'
VCAM-1	Sense	5'-TCCCTACCATTGAAGATACTGGAAA-3'
	Antisense	5'- GCTGACCAAGACGGTTGTATCTC-3'
β-actin	Sense	5'- GGACTTCGAGCAAGAGATGG -3'
	Antisense	5'- AGCACTGTGTTGGCGTACAG -3'

Table S3. LC-MS/MS conditions

System 1. ACQUITY® UPLC/Xevo® TQD [(a)] with MassLynx 4.2 software [(a)]

Column: BEH C18 (1.7 μm, 2.1 mm ID × 100 mm) [(a)] operated at 40°C

Mobile phase: (A) 0.1% (vol vol^{-1}) formic acid and (B) acetonitrile−isopropanol (1:1) with a flow rate at 0.3 mL min^{-1}

Gradient elution program:

Time (min)	0	4	10	10.1	13
%A	50	5	5	50	50
%B	50	95	95	50	50

Injection volume: 2 μL

Ionization: electrospray (positive) with a collision energy at 25 V

System 2. LC-20AD [(b)]/LTQ-Orbitrap XL™ [(c)] with Xcalibur 2.1 software [(c)]

Column: CAPCELL CORE C18 (2.7 μm, 2.1 mm ID × 750 mm) [(d)] operated at 40°C

Mobile phase: (A) 0.1% (vol vol^{-1}) formic acid and (B) acetonitrile−isopropanol (1:1) with a flow rate at 0.2 mL min^{-1}

Gradient elution program:

Time (min)	0	12	60	60.1	65
%A	50	5	5	50	50
%B	50	95	95	50	50

Injection volume: 2 μL

Ionization: electrospray (positive) with a collision energy at 35 V

System 3. Nexera system [(b)]/TripleTOF® 6600^{+} [(c)] with SCIEX OS Analyst® software [(c)]

Column: BEH C18 (1.7 μm, 2.1 mm ID × 100 mm) [(a)] operated at 40°C

Mobile phase: (A) 0.1% (vol vol^{-1}) formic acid and (B) acetonitrile−isopropanol (1:1) with a flow rate at 0.3 mL min^{-1}

Gradient elution program:

Time (min)	0	4	10	10.1	13
%A	50	5	5	50	50
%B	50	95	95	50	50

Injection volume: 2 μL

Ionization: DuoSpray™ electrospray (positive) with a collision energy at 30 V

System 4. ACQUITY® UPLC/Xevo® TQD [(a)] with MassLynx 4.2 software [(a)]

Column: BEH C18 (1.7 μm, 2.1 mm ID × 50 mm) [(a)] operated at 40°C

Mobile phase: (A) 0.0025% (vol vol^{-1}) formic acid and (B) acetonitrile containing 0.0025% (vol vol^{-1}) formic acid with a flow rate at 0.2 mL min^{-1}

Gradient elution program:

Time (min)	0	0.5	4	10	10.1	13
%A	80	80	5	5	80	80
%B	20	20	95	95	20	20

Injection volume: 10 μL

Ionization: electrospray (positive) with the following multiple reaction monitoring settings:

Analyte	Precursor ion (*m*/*z*)	Product ion (*m*/*z*)	Cone voltage (V)	Collision energy (V)
SEA [(f)]	328.6	62.2	10	21
OEA [(g)]	326.3	62.2	10	21
LEA	324.5	62.2	10	21
AEA	348.3	62.2	10	30

System 5. Nexera system [(b)]/LCMS-8050 [(b)] with LabSolutions Insight software [(b)]

Column: YMC-Triart C8 Metal-Free PEEK-Linked Column (1.9 μm, 2.1 mm ID × 100 mm) [(a)] operated at 50°C

Mobile phase: (A) methanol/acetonitrile/water (1/1/3 by volume) containing 5 mM ammonium acetate and 0.025% (w w^{-1}) ammonia water and (B) 2-propanol containing 5 mM ammonium acetate and 0.025% (w w^{-1}) ammonia water with a flow rate at 0.25 mL min^{-1}

Gradient elution program:

Time (min)	0	3	8	8.1	12
%A	80	2	2	80	80
%B	20	98	98	20	20

Injection volume: 2 μL

Ionization: electrospray (positive) with the following multiple reaction monitoring settings:

Analyte	Precursor ion (*m*/*z*)	Product ion (*m*/*z*)	Q1 Pre Bias (V)	CE (V)	Q3 Pre Bias (V)
p-OEA	406.40	308.35	−12	−18	−13
OEA	326.30	62.20	−15	−17	−22
p-LEA	404.40	306.28	−11	−19	−20
LEA	324.30	62.20	−17	−16	−24
p-AEA	428.35	330.26	−17	−15	−20
AEA	348.30	62.25	−17	−16	−23
p-HEA	394.30	296.50	−20	−16	−18
HEA	314.30	62.30	−23	−17	−24

[(a)] Waters, Milford, MA, USA; [(b)]Shimadzu, Kyoto, Japan; [(c)]Thermo Fisher Scientific, Waltham, MA, USA; [(d)]Osaka Soda, Osaka, Japan; [(e)] AB Sciex, Tokyo, Japan; [(f)] stearoyl ethanolamide; [(e)] oleoyl ethanolamide.

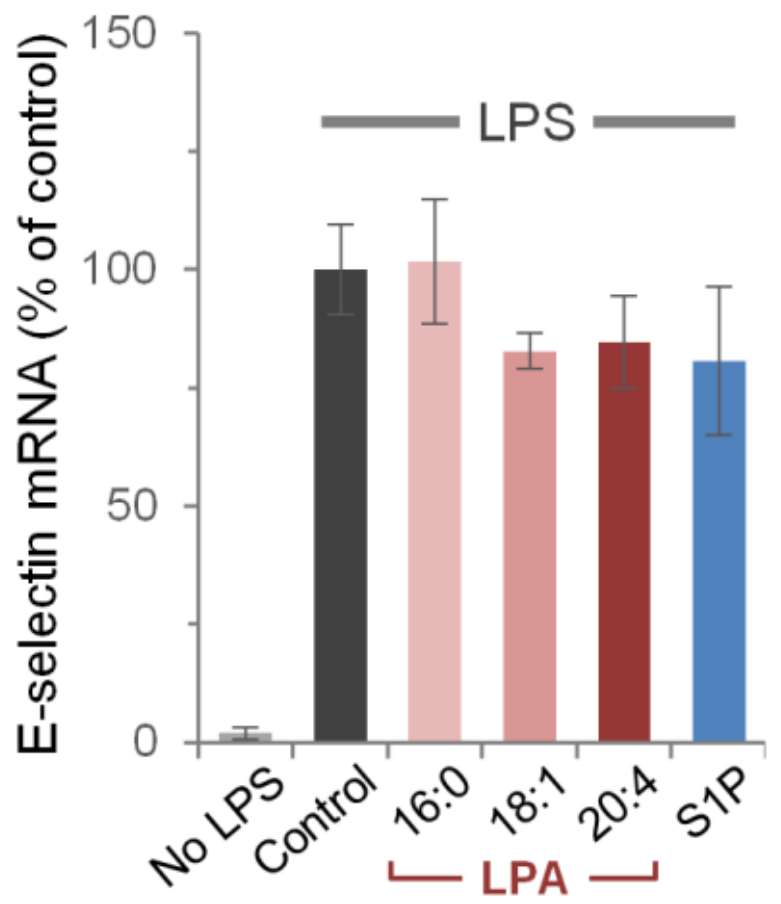

Figure S1. LPA and S1P lack the activity to suppress HMVEC activation. (A) Effects of LPA species with different acyl chains (palmitoyl: 16:0, oleoyl: 18:1, and arachidonoyl: 20:4) and the major S1P species, S1P(d18:1), on the LPS-induced E-selectin expression in HMVECs. Each value represents the mean ± SD from triplicate determinations.

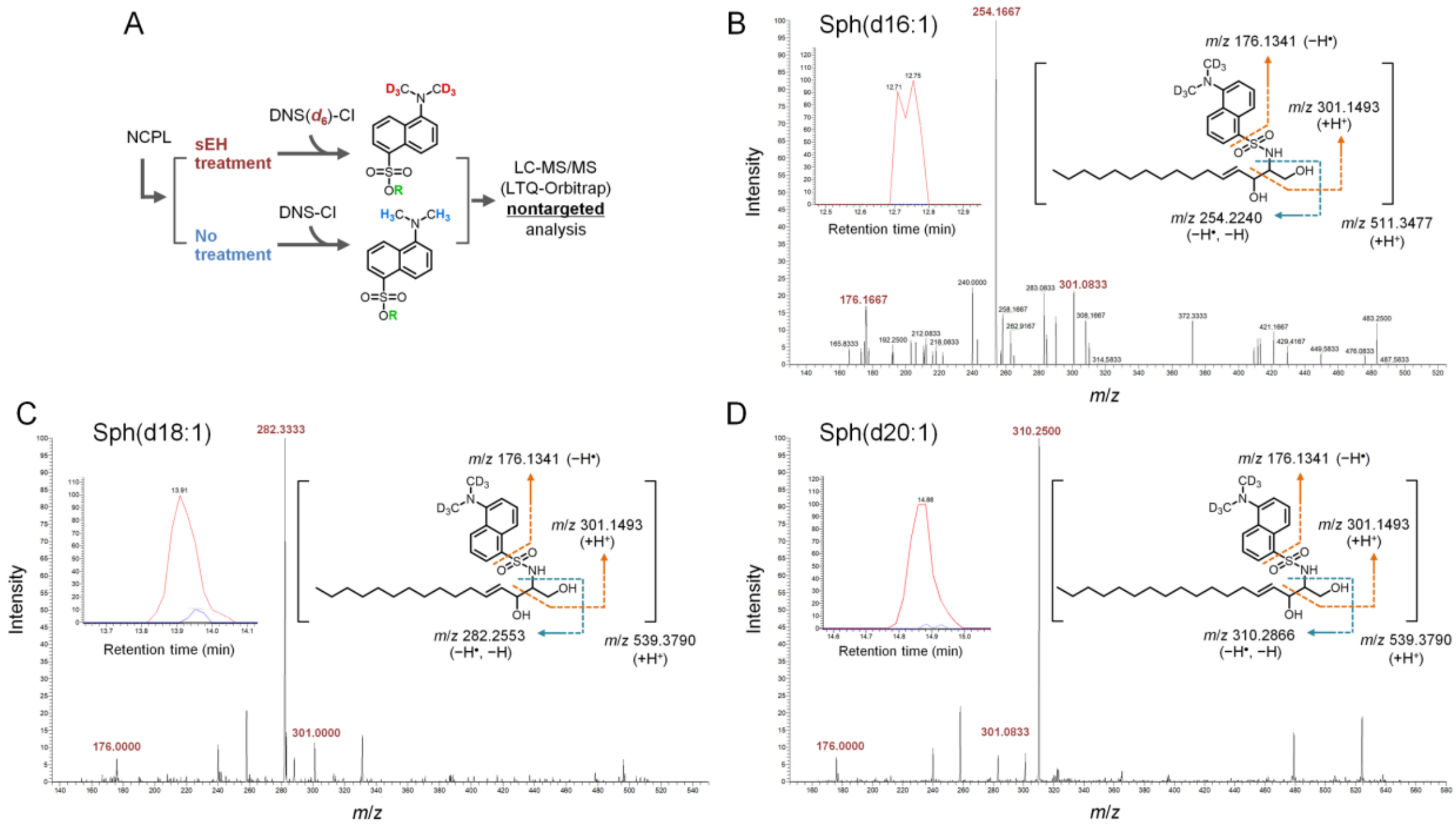

Figure S2. A nontargeted exploration of N-phos substrates identifies minor S1P species. (A) Outlines of the experimental strategy in which an LTQ-Orbitrap spectrometer was used to identify trace amounts of unknown N-phos products. (B−D) Mass chromatogram and spectrum of the potential products. Possible assignments for the daughter ion peaks are shown. These results suggest that the products are sphingosine(d16:1), sphingosine (d18:1), and sphingosine (d20:1). Sphingosine(d16:1) and sphingosine (d20:1) are atypical species present in less than 1/10 of sphingosine(d18:1) in mammalian tissues. Sph, sphingosine.
